# Supplementary material for: Active site specificity profiling datasets of matrix metalloproteinases (MMPs) 1, 2, 3, 7, 8, 9, 12, 13 and 14
Source: Data Brief. 2016 Feb 22;7:299–310. doi: 10.1016/j.dib.2016.02.036 (PMC4777984; doi:10.1016/j.dib.2016.02.036)
Supplement: Supplementary file 10 — Supplementary material [file mmc10.zip › WebPICS_hMMP8_G_1%/P3prime.html]

 

PICS results


|  |  |
| --- | --- |
| **P3prime\_A**  26 in 120 sites   21.7 %    effects > 10 perc. pnts.   (vice-versa in brackets)  P3\_I: 15.6 (45.0) |  |
| **P3prime\_G**  29 in 120 sites   24.2 %    effects > 10 perc. pnts.   (vice-versa in brackets)  P1\_A: 13.3 (29.6)   P2prime\_K: 17.1 (19.8) |  |
| **P3prime\_N**  10 in 120 sites   8.3 %    effects > 10 perc. pnts.   (vice-versa in brackets)  P2\_F: 22.5 (25.0)   P1prime\_V: 20.0 (16.7)   P2prime\_I: 29.2 (22.5) |  |
| **P3prime\_Q**  7 in 120 sites   5.8 %    effects > 10 perc. pnts.   (vice-versa in brackets)  P2\_H: 11.8 (27.5)   P2\_Q: 32.9 (19.2) |  |
| **P3prime\_S**  12 in 120 sites   10.0 %    effects > 10 perc. pnts.   (vice-versa in brackets)  P2\_A: 13.3 (11.4) |  |
| **P3prime\_V**  10 in 120 sites   8.3 %    effects > 10 perc. pnts.   (vice-versa in brackets)  P3\_H: 25.8 (51.7)   P2\_W: 26.7 (66.7)   P1\_P: 22.5 (25.0)   P1prime\_C: 14.2 (20.3)   P1prime\_F: 24.2 (34.6)   P2prime\_Q: 30.0 (25.0) |  |
